# Keeping our Research Plumb: Theory-Driven Design and Analysis for the Study of Instructor Epistemologies

**DOI:** 10.1021/acs.jchemed.5c00852

**Published:** 2025-11-11

**Authors:** Nicole C. Greco, Rosemary S. Russ, Ryan L. Stowe

**Affiliations:** † Department of Chemistry, 5228 University of Wisconsin—Madison, Madison, Wisconsin 53706, United States; ‡ Department of Curriculum and Instruction, University of Wisconsin—Madison, Madison, Wisconsin 53706, United States

**Keywords:** Research, General Public, Chemical Education Research, Assessment, Learning Theories

## Abstract

The field of chemistry education is increasingly interested in thinking beyond what content our courses explicitly teach and toward thinking about implicit ways of knowing and learning (i.e., epistemologies) our courses elevate. Previous scholarship gives us reason to believe that studying epistemologies in undergraduate chemistry will provide us with important insight into how students navigate and learn in our courses. However, we are only beginning to explore the ways in which instructors’ goals for knowing and learning shape (or not) students’ experiences in our courses. If we want to make progress in understanding student and instructor epistemologies, then we must design research in a way that matches our theoretical model of its ontology. To assist the community in considering how theory might drive design and analysis in studies related to epistemology, we trace decisions we made while exploring the dynamics underlying instructors’ epistemology around assessment. This coherence-seeking journey illustrates that alignment between theoretical commitments and study activities is far from straightforward. Additionally, our experiences suggest that it may be generative for members of our community to unpack assumptions undergirding their theoretical framework and explore how these show up across study activities.

## Graphical abstract

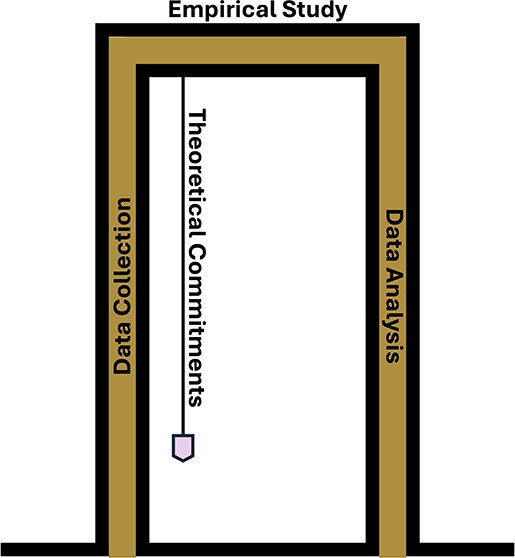

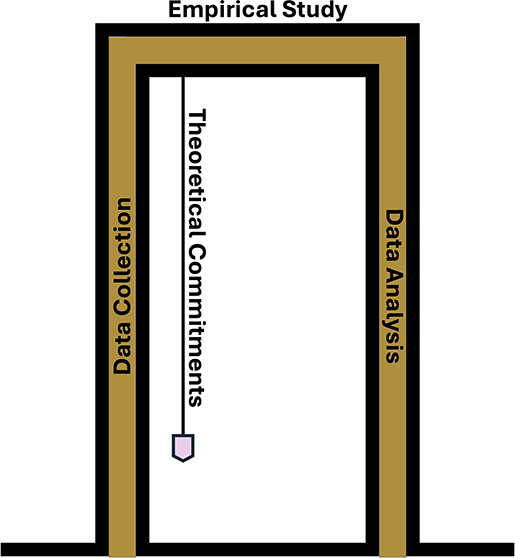

## Introduction

The field of chemistry education is increasingly interested in thinking beyond what content our courses explicitly teach and toward thinking about the implicit ways of knowing and learning our courses elevate. This shift in focus involves asking a range of kinds of questions about our courses that differ from, “What did students learn and how well did they learn it?” Instead, this focus encourages us to ask things like, “When our students take our courses, what types of knowledge do they learn are important for chemistry?” or, “What strategies for knowledge construction do they learn will allow them to be successful in chemistry?” or “What methods for justifying knowledge do they consider appropriate to this context of chemistry classrooms?”

Within science education, these sorts of questions have been studied under the construct of personal epistemology—or people’s views of knowledge and knowing.^1^ There is rich literature in that field and associated fields of scholars seeking to understand both student and instructor epistemologies as evident in K-16 science classroom learning. The focus on epistemology comes about because of a substantial body of evidence that suggests that epistemologies are consequential not only for what students learn, but also how they learn it.^2–5^

This research gives us reason to believe that studying epistemologies in undergraduate chemistry will provide us with important insight into how students navigate and learn in our courses. And indeed, it already has. For example, Sevian and Couture^6^ were able to identify common epistemic games and epistemological frames students engaged in as they solved chemistry problems, which in turn could assist teachers in recognizing how their students are approaching problem solving. As another example, Hunter et al.^7^ characterized student discourse using the structural components of the sensemaking epistemic game and inferred the epistemic form of students’ knowledge products, which provided deeper insight into how students went about making sense of chemical phenomena. Understanding how students and instructors make sense of knowledge and knowing in the chemistry classroom has provided needed texture to our accounts of the how and why of chemistry learning. It is our hope that this paper will also contribute to our understanding of epistemologies, albeit in a different way than a traditional empirical piece.

## Aligning Theory and Methods

When studying epistemologies—as in studying all constructs—we must be clear about what kind of thing epistemologies are. Consider for example the task of studying a “syllabus.” If one understands a syllabus as a standalone document meant to convey information from an instructor to a student, then one might study that document by doing some artifact analysis^8^ of it. If one understands it as a product of the cognitive labor of the instructor, one might additionally do some interviews with the instructor and then analytically compare the document analysis with the interview data. Further, if one understands a syllabus as a living document that students interpret as they go through the course, you might conduct interviews with students which you subsequently compare with the artifact and/or instructor interviews. What data you choose to collect, when and how you collect it, and how you analyze it are dependent on how you theoretically understand the construct of a “syllabus.”

In this work, we propose that the same must be true for our field’s study of epistemology. If we want to make progress in understanding student and instructor epistemologies, then we must design research in a way that matches our theoretical models of its ontology. We do not believe people will disagree with this claim; the idea that our methods should align with our theory is a hallmark of high-quality research. Chenail^9^ describes the need for our research to be “plumb,” meaning that our area of curiosity, research questions, data collection, and analytic methods are all consistent with one another. Such consistency is desirable in educational research because it helps us be confident that we have collected data appropriate to our research question and analyzed it in a way that allows us to make claims about constructs we hold to be true in the real world.

A plumb is a simple device which is a heavy metal bob attached to a string. The user holds the top of the string and the line that is created by the string with the bob hanging down shows a true and vertical straight line. For example, when hanging a door, a carpenter might use a plumb line to ensure they have a completely vertical door frame. The extent to which the frame is “out of plumb” affects whether the door will be able to open and close. Importantly for us, things can be more or less out of plumb; Chenail describes the notion of “drift” meaning how far out of plumb something is. In the case of the door, we can imagine a frame that is slightly out of plumb where the door can still close, or a frame that is highly out of plumb where the door cannot close. “Plumbness” is a continuum rather than a binary.

In this paper, we argue that it is our theoretical commitments that should set the plumb line for our research. These theoretical commitments help us to assess how far we have drifted from our assumptions and goals and help us to bring things that have drifted back into alignment. Being so “out of plumb” that our research doors cannot close is something that can happen quickly and easily when we are not explicit about aligning our theoretical commitments with the empirical aspects of our study. Importantly, the research team who designed a study is best positioned to consider the extent to which that study is plumb; individual researchers should assess plumbness from the perspective of their theoretical commitments which may or may not be explicit for those outside the research team.

While it is easy to say, “researchers should assess whether their study is aligned with their theoretical commitments,” working toward such alignment is, in fact, quite challenging. There are a host of decisions one must make when designing and carrying out a study (e.g., How should I model epistemology?; What data can I collect and how should I do so?) and virtually none of these have straightforward right answers. As such, keeping a study from drifting far out of plumb requires continuous reflection on how one's theoretical commitments show up in our study design, data analysis, implications, etc. Here, we template what such reflections might look like across the arc of a study. Specifically, we trace decisions we made while studying the dynamics underlying instructors’ epistemology around assessment. In doing so, we aim to surface the kinds of decisions one can face when studying epistemology as well as theoretically principled ways of navigating these decisions. We conclude by articulating lessons we learned while working to align our study activities with our theoretical commitments.

Working toward our goals for this paper requires a somewhat different format than what is typical for a research paper in this journal. In what follows, we will present empirical data from a research study. However, rather than presenting a theoretical framework, a methods section, then an analysis and discussion section, we will weave together these pieces to tell a narrative of how our theoretical framework shows up in each of the research choices we made. We will begin by presenting a brief ontology of epistemology as we seek to enact it in our research. We then describe the context of our study. After that, we present a series of decisions we made around data collection and describe how they follow from our chosen ontology of epistemology. We then do the same thing for our data analysis methods—highlighting how they also follow from our ontology of epistemology. We end by considering what our efforts to conduct plumb research can teach the broader community about aligning empirical work with theoretical commitments. We believe this organization, while atypical, allows us to model what plumb research on epistemology in chemistry learning can look like.

This paper should be read as a story of how we designed, used, and analyzed data from interviews to make inferences about how and why instructors assessed as they did. As we will see, navigating through this journey required us to carefully consider how our theoretical commitments manifested in our interview protocols, our analytical approach, and the implications that could reasonably be drawn from our analysis. To be clear, our goal in this paper is precisely not to claim that the data collection and analysis decisions we made are the *only* or *best* way to approach studying epistemologies. Instead, we contend that our decision points and ways of navigating these decisions have the potential to be generative to other scholars who are interested in exploring how and why people approach learning in an array of contexts.

### Our Theoretical Framework: A Resources Model of Epistemology

The important role epistemologies play in learning has led many scholars to consider useful ways of modeling epistemic cognition. Early efforts in this vein treat epistemologies as traits that students have with some traits being better/more useful than others.^10,11^ These traits were thought to slowly develop along a single trajectory from naïve to sophisticated. Later work conceptualized epistemologies as multidimensional, with dimensions developing more-or-less independently of one another.^12^ These models also assume there are context-invariant “best” ways to know and learn that students move toward over the course of years.

Here, we draw from empirical and theoretical work that demonstrates students’ epistemologies to be frequently tacit, dynamic, and context-sensitive.^13–16^ That is, in contrast to earlier developmental or dimensional models, we assume that one’s epistemologies are often unspoken, unconscious, and must be inferred from behavior, that one’s epistemologies may shift rapidly (over a period of minutes), and that the utility of particular epistemic ideas is dependent on context.^17^ Of course, these three features of the ontology of epistemology are not mutually exclusive or independent. Their tacit nature implies context dependency in and out of explicit contexts. Their dynamic nature comes about because of their context-dependency. However, in what follows we organize our decision making around these three features—acknowledging there will be some overlap in discussion.

Making sense of these features of epistemology requires us to move beyond early developmental models of epistemic cognition. Instead, we view epistemology as made up of many fine-grained “pieces” ^18–20^ and adopt Hammer and Elby’s^21^ “framework of epistemological resources [that are] smaller and more general than theories or traits.” People compile their views on knowing and learning in real time in response to features of a context, past experiences with similar contexts, etc. This constant compilation and recompilation of epistemologies allows us to explain (and anticipate) the rapid shifts described by empirical work in K-16 STEM settings.^2,16^

Importantly, while a resources model of epistemology sets our plumb line, other scholars could choose other ways to model epistemic cognition and still conduct theoretically coherent empirical work. Doing so would entail surfacing the assumptions undergirding one’s chosen model of epistemology and working to ensure one’s study design, data collection and analysis, implications, etc. are consistent with these assumptions.

### How a Resources Model Complicates Life for Researchers

Assuming that one’s epistemologies are often unspoken, dynamic, and activated differently across contexts creates challenges for the researcher. For example, epistemologies elicited in one context (e.g., a survey) may be very different from those that underlie behavior in another context (e.g., a class activity). Thus, given our stance on the ontology of epistemologies, it is unwise to simply administer a survey and infer stable, context-invariant epistemic beliefs from survey responses. To get around this challenge, scholars have argued that one should characterize so-called “practical epistemologies” by observing a behavior of interest.^17^

Inferring active epistemologies via observing behavior has several affordances. First, there is no need to approximate the context researchers are interested in—one instead directly observes this context. Second, data from observing behaviors (e.g., video, audio, field notes) is extremely rich; scholars can analyze both what is said as well as behavioral cues to infer different epistemological ways of being.^14^ Relatedly, from observations of behavior, one can potentially infer both individual- and group-level epistemic cognition and how these relate.

As powerful as observing authentic contexts can be to the study of epistemology, there are also notable drawbacks to this approach. Analyzing rich video data is very labor intensive, especially since almost any interaction can tell you something about epistemology. As a result of this, most studies of epistemic cognition employing *in situ* observations investigate relatively short time periods (i.e., a class period or less). Additionally, one must be able to observe the context of interest. This may be straightforward when observing the enactment of a lesson but becomes more challenging as moments of interest become removed from regularly scheduled class activities.

### Challenges of Studying Epistemology in Our Context

The empirical work that frames our discussion of aligning theory and methods is generative, in part, because the usual ways in which scholars study practical epistemologies were not feasible in our context. We were therefore confronted with the need to expand beyond *in situ* observations in ways that were consistent with our theoretical commitments. To understand why we could not simply observe the behaviors we were interested in, a bit of background on our study is required.

We aim to understand how and why organic chemistry instructors at a large, R1, midwestern university prompt for and evaluate students’ knowledge products on exams. Instructor participants in our study consisted of tenured professors, pretenure professors, and nontenure track professors whose appointments are teaching-focused. Participants were recruited in a manner consistent with University of Wisconsin–Madison IRB 2022–0899. All participants teach one or more large-enrollment (>200 students) organic chemistry course(s). Most students who enroll in these courses are nonchemistry STEM majors intent on a career related to engineering or healthcare. Organic chemistry instructors at this institution have substantial latitude to decide what and how they teach and assess. As such, we anticipated that assessment-related decisions would be based on participants’ own epistemologies rather than institutional constraints. All sections of organic chemistry taught by study participants allot the majority of course points to high-stakes exams (>75%). This means the exam decisions instructors made, and the epistemologies that underlied these decisions, were extremely consequential for students. Pseudonyms for study participants, their position, courses they teach, and their years of teaching experience are described in Table 1.

**Table 1:** Relevant Characteristics of Instructor Participants

Instructor	Position^a^	Courses Taught	Years of Teaching Experience^a^
James	Nontenure track	Organic I, Organic II	>10 years
Liam	Pre-Tenure	Organic I	>5 years
Mark	Tenured	Organic II	>17 years
Calvin	Tenured	Organic I	>12 years

a At time of interview.

Exploring how and why instructor participants prompt for and evaluate students’ knowledge products requires that we surface epistemologies that underlie exam-writing and exam-grading. Unfortunately, observing these behaviors was either not possible (for exam writing) or not useful (for exam grading/writing). Our study participants frequently wrote exams in scavenged moments between meetings or late in the evening. As such, we could not be in the room while exam writing was happening. In addition, both exam writing and exam grading are done largely in silence (with the exception of occasional TA questions about the rubric). This means that naturalistic observations would not tell us much. To explore instructors’ epistemic cognition around assessment, a more directed approach than pure observation was required.

## How Our Theoretical Framework Drove Data Collection

### A Case for Interviews

Since the behaviors we were interested in were both difficult to access and potentially uninformative, we turned our attention to the possibility of constructing interviews that probed instructors’ assessment-related epistemologies. Doing so opened a world of methodological possibilities as well as a host of new challenges.

Interviews are one of the most widely used qualitative methods in education and the social sciences more broadly. They allow researchers to obtain rich and nuanced information about participants’ experiences, thoughts, and perspectives.^22,23^ Further, they allow us to understand phenomena through the eyes of our participants.

Part of the value of interviews is their range and adaptability. Interviews have been used to explore PhD chemists’ sensemaking during moments of uncertainty,^24^ how the social identities of marginalized women in chemistry doctoral programs influence the development of their science identities,^25^ as well as how students use structure–property relationships to predict molecular properties.^26^ As Bryman^27^ describes, “it is the flexibility of the interview that makes it so attractive.” They can be used with a wide range of participants engaged in a wide range of activities in a wide range of contexts.

Because interviews can be used in such a broad range of studies, it is not possible to say that interviews—or specific types of interview tasks—are necessarily fully plumb. When asking questions about plumbness, we must always ask whether decisions that were made are appropriate to the theoretical conceptualizations used in the study. Thus, interviews and interview tasks can be more or less plumb depending on the theoretical commitments guiding the study. Below, we track how our theoretical commitment to a resources model of epistemology showed up in our specific approach to designing interviews for our context. Specifically, we discuss how our methods attend to the tacit, dynamic, and context-dependent nature of epistemologies.

### Consideration for the Tacit Nature of Epistemologies

If we are serious about epistemologies frequently being unconscious and unspoken, then our interview protocols cannot simply ask instructors, “Why do you assess in the way that you do?” and at the same time maintain plumbness with our theoretical commitments. Instead, we need to also build moments in which study participants engage in behaviors from which we can infer underlying epistemic ideas. For us, authentic tasks^28^ we wished instructors to engage in related to exam-writing (prompting for knowledge products) and exam-grading (evaluating knowledge products). We discuss the specifics of these tasks below. What is important for us here is that the tasks do not necessitate instructors to have conscious, explicit awareness of their thinking. Instead, by simulating exam-related contexts, we hoped to surface epistemologies that underlie important aspects of instructors’ assessment practices.

### Consideration for the Dynamic Nature of Epistemologies

Assuming that participants’ epistemologies may shift from one moment to the next makes it unreasonable to conduct a single interview and, after analyzing interview dialogue, make sweeping claims about what instructors believe. Instead, we carried out multiple interviews throughout the semester in order to (potentially) surface patterns in resource activation across time. Each participant was interviewed three times over the course of the semester (see Figure 1). The focus of the first interview was prompting for knowledge products on exams (i.e., what content is on the assessments and how they ask for it); the focus of the second interview was evaluating student knowledge products; and the focus of the third interview was reflecting on the semester and student experiences with assessments. Taken together, these interviews reflect sites where we expect instructors to make consequential decisions about what knowledge and ways of knowing deserve space on course assignments. By conducting these interviews at multiple time points, we aimed to maintain plumbness by surfacing the dynamic—and as a result, sometimes inconsistent—nature of instructor epistemologies.

**Figure 1:**
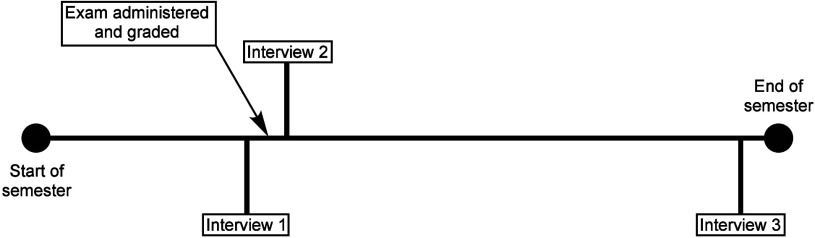
General timeline of interviews for participants. Interviews 1 and 2 were conducted approximately 1 week apart.

### Consideration for the Context Dependency of Epistemologies

Recall that we intend to use interviews to explore epistemologies underlying behavior that is difficult to observe. To do so, we must assume that we can simulate unobservable moments of interest such that participants in the interview behave in a similar manner to how they would normally engage in such tasks. That is, we want to create moments in which the set of epistemologies we would expect to be able to observe, or be able to infer from observation of behavior, would also be activated in the interview. So, if an instructor unconsciously activates epistemic resources such as *knowledge is justified by aligning with canon* and *knowledge must have certain normative structural features* when writing an exam in the dead of night, we should like them to *also* unconsciously activate these same resources when considering exam authoring in an interview.

We claim that it is, in-principle, possible to create interview conditions for tacit activation of epistemological resources^29^ (i.e., “contexts”) that overlap with similar conditions encountered in an inaccessible context (see Figure 2). Note that we do not claim that *all* contexts one might observe may be simulated in an interview—this is why Figure 2 shows some “observation contexts” that do not overlap with “interview contexts.” It may be challenging to simulate the context of skydiving in an interview room, for example. However, we claim that there is some overlap and that this overlap may be useful to researchers.

**Figure 2:**
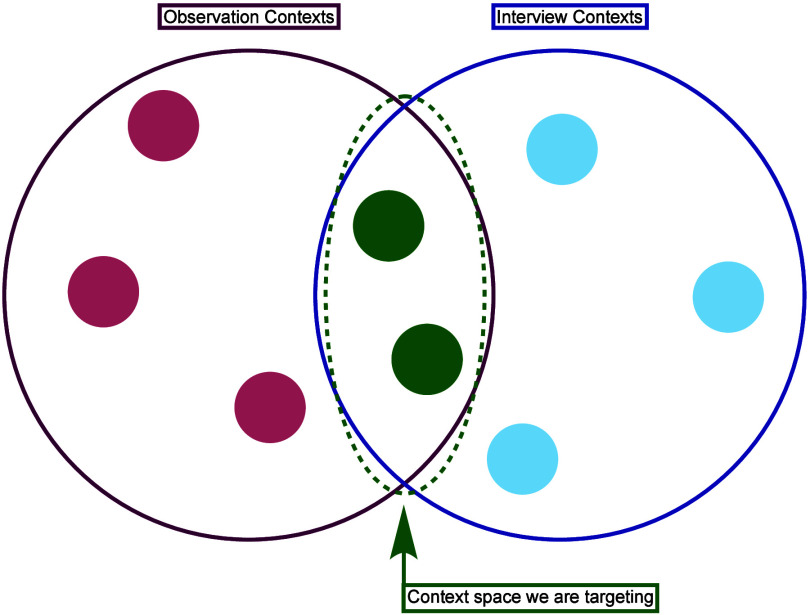
Venn diagram describing the hypothesized overlap between observational contexts and contexts that can be simulated in an interview.

Conversely, we think it is likely that some interview contexts are rarely encountered naturally—we have never been asked, “Is there a difference between a scientific theory and a scientific law?” (a common type of question on Nature of Science surveys)^30^ outside of a contrived context like a research questionnaire, for example. Such interview-only contexts are represented by the light blue circles in Figure 2. Again, we wish to highlight that these questions are merely inconsistent with our theoretical commitments and would thus cause some drift in our specific study; they may be perfectly plumb with the commitments of others.

If we allow for the existence of interview contexts that are experienced by participants as analogous to professional activities, then is it reasonable to ask: How do we choose which professional contexts to simulate? Our answer to this is 2-fold. First, we should pick contexts that we have the potential to simulate. That is, we should consider how or whether features of an interview context (e.g., protocol structure, paraverbal cues from the interview) can be made similar to features of a professional context. Second, we should aim to approximate contexts that are consequential to our research and teaching objectives. In our case, the contexts of exam-writing and exam-grading are consequential to our research objectives.

Our answers to, “How should we choose which professional contexts to simulate?” led us to build protocols to approximate three contexts: 1) writing an exam and the corresponding answer key; 2) responding to student answers to exam prompts; and 3) answering survey-like questions about goals for teaching and assessment. While we have already mentioned our interest in contexts #1 and #2, context #3 may seem like an odd choice. We chose context #3 to help us explore a common practice in our field. Specifically, we regularly see researchers ask questions like, “Why do you give assessments?”^31^ and we wished to investigate how or whether epistemological ideas surfaced by such questions were consistent with epistemologies observed when instructors attempted authentic tasks.^28^ We describe how we approached designing interviews to simulate each context below.

### Surfacing Epistemologies Underlying Exam Writing

We expect that exam-writing involves an internal dialogue in which instructors iteratively check whether candidate questions are consistent with some set of criteria.^32^ Criteria may be externally imposed (e.g., content coverage expectations) or emerge from commitments about worthwhile ways to know and learn. Our interview protocol is an attempt to surface potentially tacit criteria that guided task construction. To do so, we first collected drafts of upcoming exams written by each instructor participant. We then chose focal questions from these drafts that represented task types common on organic chemistry exams (e.g., predict the product, mechanism drawing) or that appeared substantially different from these typical task types. We asked instructors to reflect on why different question types were asked, what knowledge and skills would be useful in addressing this question, and where instructors expected these knowledge and skills to come from.

Exploration of epistemologies underlying instructor-authored answer keys focused on the same set of focal questions described previously. We examined the authorized answers instructors outlined for each prompt for tacit messages about useful ways to know and learn. For example, Figure 3 suggests that authorized knowledge must include certain disciplinary jargon. We then built interview questions to prompt reflection on answer key-embedded epistemological messages.^33^ Prompts built around the key shown in Figure 3 might ask participants why underlined words were especially important, where students should get these words, and what, if any, alternative verbiage might be credit-earning.

**Figure 3:**
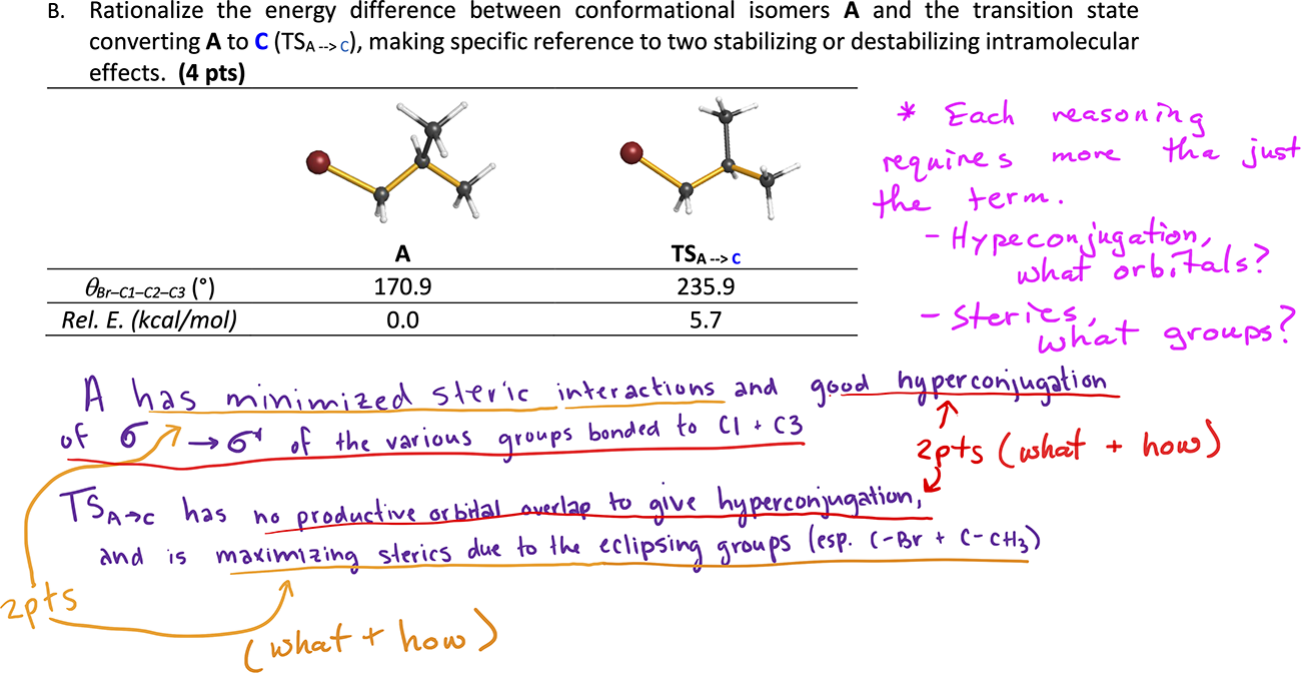
An exam answer key that suggests a specific structure for authorized knowledge.

### Surfacing Epistemologies Underlying Exam Grading

To surface epistemologies that underlie instructors’ evaluations of student knowledge products, we built several sample student answers to each of the aforementioned focal prompts. These sample student answers were meant to challenge some of the explicitly stated personal epistemologies expressed by instructors in the first interview. Doing so opened space for us to observe how (or whether) instructors’ active epistemologies were consistent across interview contexts. The research team's experiences as teaching assistants, instructors, and graders for organic chemistry courses were leveraged to ensure sample answers were fair approximations of student thinking. The use of sample student answers to simulate the contexts of grading as opposed to observing instructors grading their own students’ exams had the affordance of letting us target specific things that may not have been seen in real student answers as well as not disrupting the actual grading process for instructors by asking questions throughout (as would be the case in a think-aloud protocol^34^). An example of a constructed sample student answer is shown above in Figure 4.

**Figure 4:**
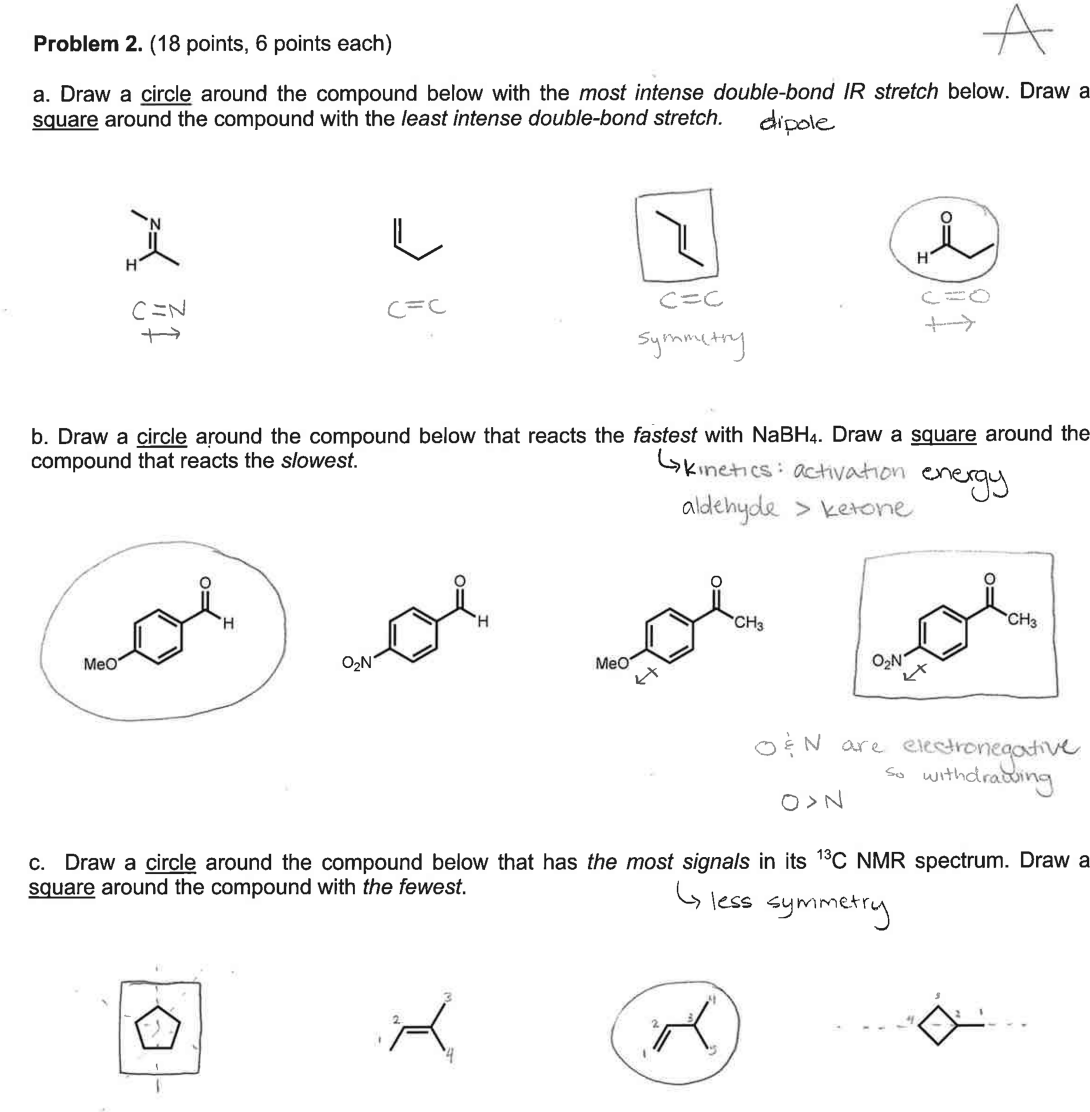
Sample student answer constructed by the research team from Mark’s second exam.

The example response shown in Figure 4 included margin notes describing student thinking. Reasoning was not prompted by this task, but several instructors stated that they value seeing how students arrived at a particular answer. Opening space for instructors to reflect on margin notes that made student thinking visible was our way of exploring how or whether explicitly stated values lined up with observed behaviors.

### Surfacing Epistemologies Underlying Responding to Survey Questions

As noted earlier, it is relatively common in our field for researchers to infer stable beliefs about teaching and learning from instructors’ responses to questions such as, “Why do you give assessments?”^31^ Doing so rests on the assumption that epistemologies consciously surfaced by such questions map onto epistemologies that underlie behaviors in naturalistic settings. In-line with work on practical epistemologies,^17^ we suggest that study participants may experience responding to survey-like questions as a context that is meaningfully different from behaviors that matter to researchers (exam writing and grading, in our case). This may lead participants to tell their interviewer an idealized response that does not correspond to activities that occur outside the interview room. To probe how/whether our efforts to simulate assessment contexts are experienced as different from survey-like questions, we included several such questions on our first interview protocol (e.g., “How do you define success in your course?”; “What is the purpose of administering assessments?”). During our analysis, we paid special attention to how (or whether) epistemologies varied between responses to survey-like questions and responses to questions meant to simulate authentic tasks.^28^

## How Our Theoretical Framework Drove Data Analysis

### Specific Analytic Scheme

We turn now to unpacking the ways in which our commitment to a resources model of epistemology informed our approach to analyzing data surfaced during interviews. Doing so will require us to adopt a vocabulary for talking about different types of epistemic ideas. While many scholars have described multiple dimensions of epistemology,^1,12,35^ here we make use of Chinn and colleagues’^36^ AIR model of epistemic cognition. We do so for several reasons: 1) Chinn and colleagues’ model synthesizes many insights from education and philosophy; 2) Chinn and colleagues’ model contains a manageable number of dimensions; and 3) we have made use of the AIR model previously and this model seems intuitive to us.^37^ Brief descriptions of each dimension of the AIR model, along with examples, are provided in Table 2.

**Table 2:** Different Dimensions of Epistemic Cognition in the AIR Model

Component	Definition	Examples
Epistemic Aims and Value	(Aim) the ends to which other aspects of epistemic cognition are directed (Value) Perceived worth of the epistemic aim	“. . .show how the scientific methods applied in problem solving and simple things like driving them toward an experimental outcome. . .that could have a result that would falsify or confirm a hypothesis. That’s what I’m trying to get them toward anyway.” (*Calvin, Interview 1*)
Epistemic Ideals	The criteria or standards that must be met for one to judge that their epistemic ends have been achieved	“Compare [some theoretical concept] to what they got in the actual data and then have them describe in some fashion whether this is consistent with the data or not.” (*James, Interview 1*)
Reliable Processes	Cognitions related to reliable and unreliable ways for achieving epistemic aims	“[Students] still need to do the work to work the problem and figure out how to apply the facts that they’ve learned to solving problems.” (*Mark, Interview 1*)

Attending to multiple dimensions of epistemology allowed us to see our theoretical commitments in our data. For example, attending line-by-line to transcript-embedded epistemic aims, ideals, and reliable processes showed both where instructors’ epistemic cognition shifted and the substance of what changed. Observed shifts and continuities aided us in selecting exemplars that appear in the discussion that follows. Additionally, the language of AIR lets us theorize about how contextual differences relate to shifts in some epistemic dimensions but not others. Perhaps, for example, an instructor maintains focus on the worth of a particular epistemic aim but shifts the success criteria (epistemic ideals) useful for achieving this aim as they move from exam writing to exam grading. If we see this, we might attempt to understand what features of each context made the different success criteria seem salient.

Our discussion of how our theoretical commitments show up in our data analysis approach is organized around the three assumptions underpinning a resources model of epistemology discussed earlier: 1) ideas about knowing and learning are frequently tacit and unconscious; 2) epistemologies are dynamic and may change on the time scale of minutes; and 3) epistemologies are context-dependent. As noted earlier, we recognize that these assumptions are to some degree interdependent (e.g., we see dynamic activation of epistemologies because participants experience a shift in context). Some filler words have been removed for ease of reading and any use of bolding within participant quotes is the emphasis of the authors and not necessarily the speaker.

### Addressing the Tacit Nature of Epistemologies

Informed by theoretical and empirical work demonstrating that epistemologies are often tacit and unconscious,^21^ our analysis attended to both explicit talk about knowing and learning (e.g., “students should support their claim with evidence”) and moments when we could infer epistemic ideas from speech about other topics. Here, line-by-line coding using AIR dimensions enabled us to construct memos^38^ describing what dimensions of epistemology underlaid what remarks about other topics. The research team could then discuss the extent to which these inferences were reasonable. To see what this looked like in-practice, consider Liam’s reflection on a decision he made to edit an assessment item while drafting his second exam.

I changed this problem at the last minute. I originally had what was, I think, in some ways a better question. . .**I kind of regret it** because what I’ve tried to do philosophically I think is—like I said a couple of times, I kind of accept that **you can succeed in different ways**. But I think finding some ways. . . where you kind of **nickel and dime people for doing the fully rote approach** instead of a more “structure/reactivity” approach, it is a way that you do not just devastate people but you can still get a little bit of information that way. . .I believe [the original question] would’ve **given me a little more information about how people are approaching the problem**.

Here, we see Liam reflecting on his decision to remove a layer of nuance from a multiple-choice question that he feared might be “too much of a trap.” He appears to be second-guessing his changes to this question because the prior iteration would “give [him] a little more information about how people are approaching the problem.” He cares about this information because he wishes to ensure his course supports engagement in certain reliable processes, such as casual mechanistic reasoning. Other reliable processes, such as memorization, are less important to Liam. As such, he sometimes tries to “nickel and dime people for doing the fully rote approach” instead of an approach that relates a molecule’s structure to observed reactivity. This explicit hierarchy of reliable processes suggests tacit information about a hierarchy of epistemic aims; Liam places more value in the epistemic aim of a nuanced understanding of structure and reactivity relationships than the epistemic aim of compiled heuristics for earning credit on rote questions.

We see further evidence of tacit epistemic ideas underlying speech about other topics in James’ reflection on how he constructed a particularly challenging exam problem:

I ran this reaction. I didn’t know what the outcome was gonna be until I got the data. I looked at the data and went, “Oh look, it di-nitrated and look, there’s a little bit of the mono-nitrate. Great.” We were hoping students would see that part and sort of **go through the authentic experience that I had while collecting the data**. . .I’d say about a quarter of the students weren't able to figure that out, which was a little bit surprising to me and really indicated that **they didn't see the data as useful to understanding** the rates of reaction, which was sort of baffling. . . The question wanted them to, based on the data, tell me which was more activating or deactivating.

His choice to collect experimental data, which he incorporated into his exam so that students would have an “authentic experience,” provides us with insight into an epistemic aim he sought to emphasize (a data-supported argument), an epistemic ideal for this aim (consistency with experimental evidence), as well as a reliable process he hoped students would engage in (looking for consistency between data and claims). Additionally, he implied this epistemic aim was worthwhile because it is “authentic” to what he/other scientists do in their professional lives.

These examples demonstrate the possibility and usefulness of describing epistemic ideas underlying speech about other topics. Specifically, they show snapshots of how instructor reflections contextualized by exam writing/grading – even reflections that do not explicitly refer to knowing and learning – gave us information about how and why these instructors assessed in the ways that they did.

### Addressing the Dynamic Nature of Epistemologies

Adopting a resources model of epistemology means our analytic choices need to be able to account for possible changes in epistemologies from one moment to the next. We therefore coded transcript-embedded epistemic aims, ideals, and reliable processes line-by-line.^39^ Doing so let us see when and how instructors’ epistemic cognition shifted within and between interviews. To illustrate how our analysis surfaced such shifts, we present an example of observed epistemic dynamicity from our second interview with Calvin.

Observed dynamics in Calvin’s epistemic cognition all relate to the assessment item shown in Figure 5. This item asks students to provide a single starting material that could be used in an experiment to infer whether an S_N_1 reaction or S_N_2 reaction occurred based on the products obtained.

**Figure 5:**
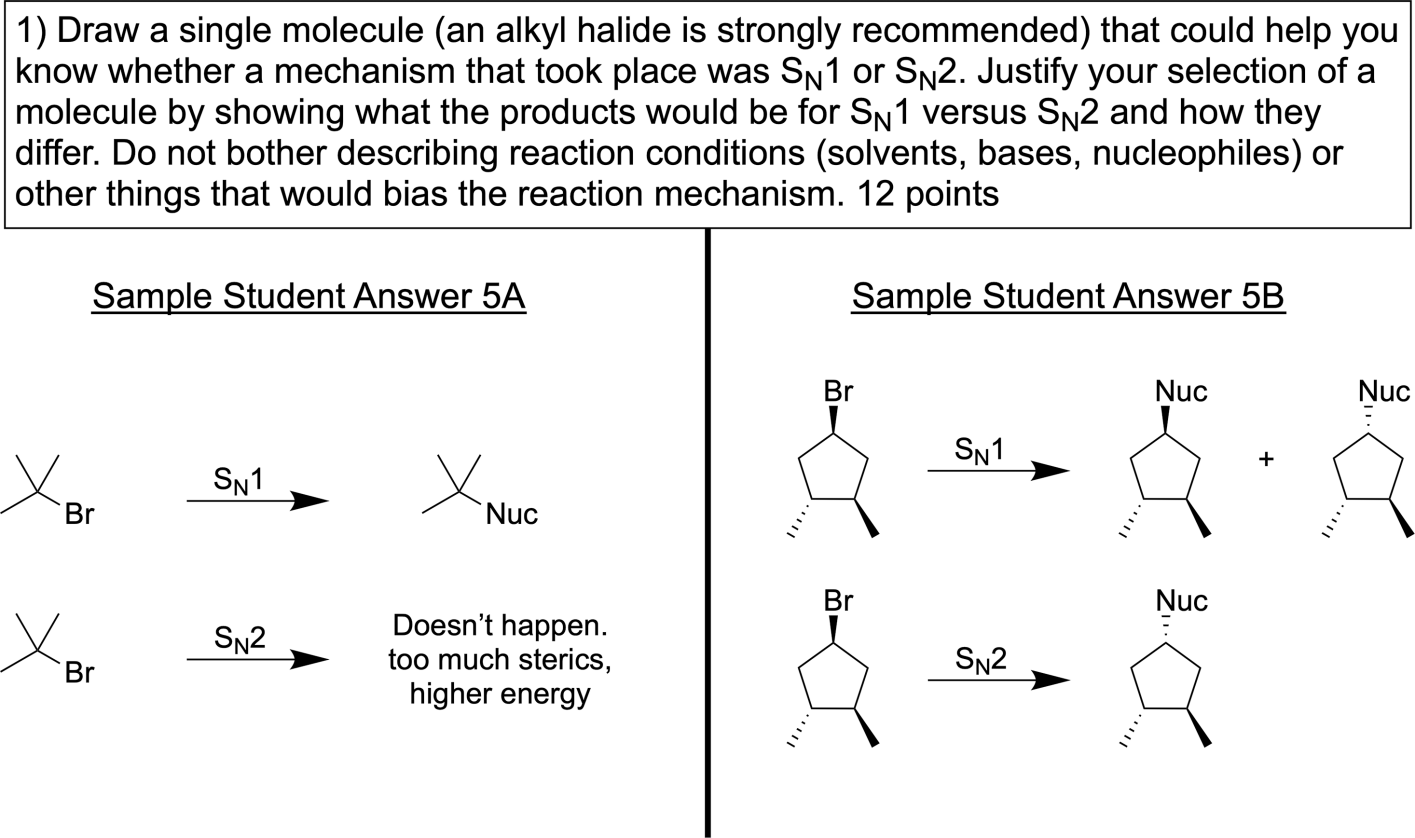
Assessment item from Calvin’s exam along with renderings of two of the sample student answers provided to him for evaluation.

Calvin describes his approach to grading the problem shown in Figure 5 as follows:

. . .if the reactant that they chose could have feasibly been used, even if they didn’t use it correctly, like **if you and I as trained chemists would be able to do it**, then they received credit for that. My rationale for that was that they might very well know what can differentiate, or could be used, but they’re not good atdrawing the products of these yet. Then it was essentially partly just for symmetry in grading then, so one third of the points for the reactant, one third of the points for the S_N_1 product. . .one third of the points for the S_N_2 product. . .**They had to be different also, otherwise there would be no point** to the answer.

Several epistemic ideals surfaced when Calvin was reflecting on how he would grade responses to this prompt. First, we see him express that good knowledge should be sensible to a professional chemist when he says, “if you and I as trained chemists would be able to [infer mechanistic information from that experiment], then they received credit for that.” Calvin then elaborates his expectations for the structure of an appropriate response. Specifically, students should indicate a reactant and different products that would be produced by each candidate reaction mechanism.

During the simulated grading task, Calvin was shown a sample student answer that perfectly mapped onto his answer key, a sample student answer that incorporated a starting material that could only react via one mechanistic pathway, and a sample student answer in which the same product would be produced regardless of whether S_N_1 or S_N_2 mechanisms were operative. Calvin’s evaluation of the second and third student responses (shown in Figure 5 as 5A and 5B, respectively) show activation of different epistemic ideals. We briefly unpack these differences here to show how our analytic approach accounted for dynamic activation of epistemological resources.

The purpose of the prompt shown in Figure 5, as described by Calvin, was to see “whether or not [students] understand how the conclusions about these mechanisms were drawn in the first place.” We built several sample student responses that attempted to embody this goal to varying extents. Sample Student Answer 5A (Figure 5) was constructed to convey that if this particular starting molecule were used, the S_N_2 conditions would not yield appreciable product (i.e., starting material would be recovered). Calvin did not want students to take this design approach as evident in his evaluation of Sample Student Answer 5A:

So, they’ve drawn an alkyl halide, they’re showing that it can undergo S_N_1, but that it can’t undergo S_N_2. So that’s where I think they’re not understanding what the question is asking because they’ve sort of circumvented half of it, right? So, this is conveying some understanding of the differences between S_N_1 and S_N_2, but it’s not conveying an understanding of **what it is to design meaningful experiments to test mechanisms**, right?

Calvin’s verbiage of “design meaningful experiments” indicates that there are certain epistemic ideals that must be met for an experimental design to be considered sound, and this sample student answer does not meet them. To help contextualize what Calvin might consider these ideals to be, we have included the following quote from his first interview where he discussed the purpose of this assessment item:

I want them to think about what we’re **actually able to observe as chemists**, which is not reaction mechanisms, **it’s only products**. And then from there, **what are we able to infer from those products**. . .I want to know if students are able to think about the difference in mechanisms of S_N_1 and S_N_2 and then think of ways that those mechanisms would bring about different products from the same reactant.

Using this quote to triangulate Calvin’s grading reflections suggests that he sees a lot of value in what the product of a given reaction can communicate. It then makes sense that Calvin could not see much value in Sample Student Answer 5A; this response does not indicate an S_N_2 product that would result upon treating the tertiary alkyl halide with some nucleophile. The mismatch between Calvin’s epistemic ideals and this student response shows up in a significant grading penalty:

In this case, they wouldn’t have received any points for any products because. . .they pick a reaction that wouldn’t give any products and then in the other case, the product then by default **can’t be distinguishable from a product that doesn’t exist**, so they’ve essentially lost the opportunity for any of those points. Then when we look at the starting material, the starting material could not work for this experiment actually, and so that in principle would be zero. But because they were still able to identify something with this, then **we assigned this two points** if they did this where they just said it could only do one or the other.

Despite this student response embodying a textbook experiment used to differentiate between S_N_1 and S_N_2 mechanistic pathways, it would earn only two out of a possible 12 points.

Immediately after evaluating Sample Student Answer 5A, Calvin is shown Sample Student Answer 5B (see Figure 5). This sample student answer was intentionally constructed to contain a starting material that would not work for this type of experiment—the same product would be produced by both mechanistic pathways. However, the product(s) are drawn in a manner that suggests student attention to the stereochemical inversion that is a hallmark of an S_N_2. During Calvin’s evaluation of Sample Student Answer 5B, he states:

Had there been explanation with it, I would’ve very confidently graded it, or told the grader to **award this full credit** because I think it conveys most everything that’s there. If anything, I would’ve said maybe 11 out of 12 points. . .**They’re seeing that an S**_**N**_ **1 mechanism gives you a mixture of stereochemistries at the reactive site, whereas the S**_**N**_ **2 gives you only the inversion**. The issue that they had was primarily with the starting material though, and that they picked one that coincidentally **wouldn’t give you unique products**. . .I guess what I’m seeing is that they’re trying to create that asymmetry though and just didn’t quite do that part of it correctly, but it **conveys to me that they knew to go for that**.

Calvin acknowledges the shortcomings of the sample student answer but still sees evidence of the epistemic aim of mechanistic thinking the question intended to assess. Stated differently, this student response (tacitly) was experienced as satisfying Calvin’s epistemic ideals in this moment.

Interestingly, we see a shift in Calvin’s active epistemic ideals as he evaluated the two student responses shown previously. Sample Student Answer 5A was not given full credit because the student did not depict two different products they expected to result from the two candidate reaction mechanisms. Sample Student Answer 5B received a more positive assessment despite the student drawing the same product for both S_N_1 and S_N_2 pathways. The experiments suggested would thus give exactly zero useful mechanistic information, even to “trained chemists.” Despite this shortcoming, Calvin was happy to evaluate the structure of the student’s knowledge product rather than the utility of the experiments proposed. His epistemic ideals thus shifted from “suggests an experiment that would provide mechanistic information to a trained chemist” toward “depicts stereochemical inversion (or not) at the reactive carbon”.

Our purpose with this section is not to critique Calvin’s assessment practices. Instead, we wish to demonstrate how our data analysis strategy allowed us to anticipate and describe epistemic shifts our theoretical model of epistemology would predict. By doing so, we provide evidence that our way of analyzing data was congruent with the theoretical assumption that epistemic ideas are dynamic.

### Addressing the Context-Dependency of Epistemologies

Our commitment to a resources model of epistemology leads us to anticipate that tacit epistemologies might vary as instructors move between different interview contexts (e.g., exam grading, exam writing). We already described how our design attended to this possibility and in our analysis of the data, we looked across simulated contexts to see how or whether epistemological ways of being were the same or different. Below, we describe (in)­coherences in epistemological resource activation across the simulated contexts that comprised our interview protocols. Line-by-line descriptive coding^39^ of epistemic aims, values, ideals, and reliable processes enabled us to describe each local epistemological coherence and note when each coherence shifted across time.

### An Example of Consistent Epistemic Cognition

We can use James as an example of consistent epistemological resource activation across a survey-like context (i.e., a general question about assessment), reflection on a recent assessment, and engagement in an authentic task^28^ (assessment grading). We begin with the survey-like question. When asked how he defines success in his second-semester organic chemistry course, James replied:

Uh, success in my course is a student who is **able to explain how and why chemical reactions occur**, rationalize how chemical structures lead to differences in relative energy, who can **analyze spectroscopic data to determine what happened**, and can take all of those things and **put them together in a coherent picture**, hopefully with an internal model in their head that functions to address all of the phenomena covered in this course and all future courses.

James’s explicitly stated epistemic aim was more specific than what was seen with other instructors, who talked vaguely about “critical thinking” or “problem solving skills.” This clarity of purpose helped paint a clear picture of how James conceptualizes knowledge and learning within his course: engaging in analysis of authentic data (reliable process) to create a model (epistemic aim) that aligns with spectroscopic evidence and is coherent with prior knowledge (epistemic ideals).

Shortly after explicitly articulating his hoped-for epistemic aims, ideals, and reliable processes, when reflecting on an assessment he wrote, James was asked *why* he built extended prompts around spectroscopic and computational data:

Oh, because **I want authentic data to be the driver** rather than assumptions about what happened. I’m not asking them to rely on memorized patterns or preconceptions or possibly really good understandings they have about similar systems. . .Um, I wanted them to confirm that there was an outcome and **this is how chemists authentically confirm that there are outcomes**. They get the spectra, they analyze the spectra, and then they decide what’s taken place and making the data fit to the assumption. . .and so here, the focus first is on **what does the data actually tell you** what has actually occurred, and then we ask about **why did that occur and what does that mean**?

Here, James highlights how his assessment tasks were designed to emphasize and reward construction of evidence-based explanations and models. He hopes students will approach such tasks by iteratively searching for consistency between claims and provided spectroscopic evidence. Looking across this and the prior quote, we see James consistently activating the same epistemic aims (models or explanations), ideals (alignment with data) and reliable processes (iteratively checking for consistency between claims and data). We suspect this consistency is due, at least in part, to James’s work to clarify what epistemologies he values for students and how those might be communicated by the structure of what he builds.

The research team anticipated that “consistency with data” might be one of a collection of epistemic ideals James activated as he wrote and graded exams. To probe the relative importance of this ideal, we constructed a sample student answer that did not properly interpret the provided data but was otherwise internally consistent. We hoped James’s engagement with this answer would tell us how he prioritized “consistency with data” relative to “internally consistent” in-the-moment. During his evaluation of this sample student answer, James did not focus on the internal consistency of the response but rather continued to prioritize the epistemic ideal of alignment with data as indicated by the following quote.

Um, yeah. [Part K is] **a lot harder to award credit for** because it’s basing everything on a bad assumption; **it’s basing it on a misinterpretation of the data** [in part J]. There’s not a whole lot that’s actually right here. . .My assumption based on the written work [in parts K and L] is **they just made an assumption and jammed everything into it**.

This quote, and the two that preceded it, suggest James consistently activated aims, ideals, and reliable processes across several interview contexts. He continued to favor the reliable process of aligning with data and not (just) theoretical assumptions to achieve the high-value epistemic aim of an evidence-based model for a chemical reaction. Sincethe sample student answer did not properly interpret the data, the assessment answer key did not allow for significant partial credit for activating unreliable processes.

### An Example of Inconsistent Epistemic Cognition

A key analytic affordance of a resources model of epistemology is that it can account for both observed stability in resource activation—which we saw with James—and observed instability, which we will explore now. Specifically, we highlight dynamics in Calvin’s activation of epistemic resources as he answered explicit questions about his epistemological goals for students and subsequently graded student responses to an exam prompt.

As with James, we will begin exploring Calvin’s epistemic cognition by considering his response to a general, survey-type question. When Calvin was asked about the types of knowledge and skills he assesses in his first-semester organic chemistry course, he responded:

The [exam] problems that I think more about and interpret more from are ones that are more about problem solving and critical thinking. Um, some of my favorites are things like asking students to—so once we’ve taught a concept, I then would ask them a question that asks them to **design an experiment**, like “pick the reactant,” for example, that would allow them to have **arrived at the same conclusions and knowledge that chemists did before** to try and evaluate whether or not they’re seeing the sort of the problem solving process in that, the **scientific method being applied in comparison with the pattern recognition**, right?

Here, we see Calvin activate a similar epistemic aim to what James articulated: students should “think like a chemist” by critically reviewing their own hypotheses to arrive at canonical conclusions. This aim was common across all instructors whom we interviewed.

Unlike James, however, we see a shift in Calvin’s epistemologies as he engages in grading a sample student response to an exam prompt. This student response (see Figure 6) was constructed to approximate a student trying to rationalize their way through a mechanism—at least as much as a one-way assessment can allow—with questions written in the margins. It represents our attempt to show what it might look like for a student to engage in the chemist-like reasoning Calvin purports to value while also expressing some non-normative ideas.

**Figure 6:**
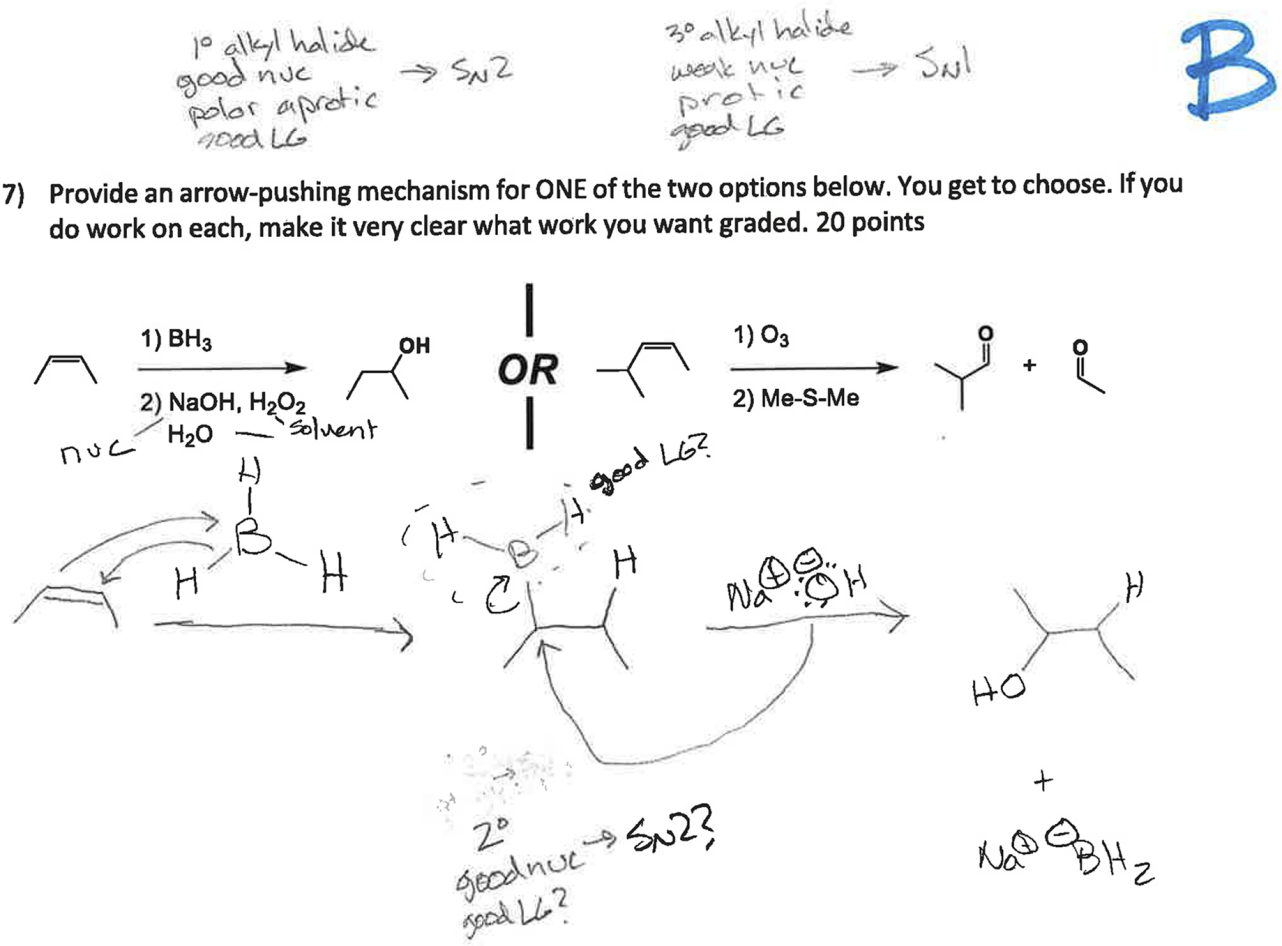
Sample student response to a prompt on Calvin’s exam.

While evaluating the response in Figure 6, Calvin said:

So, **I see some actual, like reasonably meritorious thinking here**. Um, **it might have even been a mechanism that chemists had to ponder at one point**, honestly. But, um, **because the actual mechanism is presented so, so cleanly** for all students [in lecture], I wouldn’t have given any more points than [four out of 20].

This self-narration of Calvin’s impressions of the sample student answer suggests that the reproduction of a canonically correct mechanism was the only epistemic aim he valued while grading this question. Note that Calvin expressed awareness of other epistemic aims—such as plausible mechanisms that are “reasonably meritorious”—but he chooses to place less importance on these due to “the actual mechanism [being] presented so cleanly [in lecture].”

Inconsistencies such as those observed in Calvin’s dialogue are expected and explainable if we take up a resources model of epistemic cognition. It is possible, for example, that Calvin experienced answering questions about epistemic aims for students differently from our attempts to simulate exam grading. Perhaps questions about epistemic aims cue aspirational ideas about how he would like chemistry students to know/learn while engagement in exam grading brings to mind a host of logistical concerns (e.g., ease of grading, time it takes to grade) that act to constrain available epistemologies. Further empirical and theoretical work will explore these possibilities.

The choice to take a fine-grained, multidimensional approach when analyzing transcripts aligns with the theoretical assumption that epistemologies are context-sensitive and allowed us to identify and map out local coherences and inconsistencies in our data. Using the AIR model while doing so enabled us to describe the substance of epistemic ideas that were (in)­coherent. These careful descriptions let our team come to agreement about what changed when across our data set. Stated more generally, attending to both substance (i.e., AIR dimensions) and temporality (when shifts occurred) helped ensure that our analysis remained plumb with our theoretical commitments.

## Implications and Conclusion

This research journal, like all education research journals, requires submitted studies to clearly articulate the theoretical framework under which the research operates and the methods the study employed. It is not, however, always explicitly discussed how those theoretical commitments manifest within study design and analysis decisions. Glossing over how theory is used as the “plumb line” for a study^9^ risks design and enactment of incoherent studies that cannot support useful implications for teaching and learning. As such, alignment of theory and methods is crucial to conducting high-quality research and producing meaningful results. Here, we describe in-detail decisions made to work toward a theoretically coherent study of instructors’ epistemic cognition around assessment. In doing so, we seek to concretely demonstrate what achieving the more general goal of aligning theory and methods might look like in-practice.

So, what can our coherence-seeking journey teach members of the chemistry education community, many of whom may have different theoretical commitments than us? There are three lessons we have learned that, we claim, are useful regardless of one’s theory of choice:

### 1 Working toward plumb research requires unpacking assumptions underlying one’s chosen theoretical framework

Theoretical frameworks are complex, and it can be challenging to know which aspects of a given framework are salient across the arc of a study. We found it useful to unpack the assumptions about the nature and dynamics of epistemic ideas embedded in the resources model of epistemology (i.e., epistemic ideas are often tacit, dynamic, and have varying utility depending on context). Doing so allowed us to frame specific questions that we could answer as we designed our study, analyzed our data, and drew implications from this analysis. For example, when analyzing our data, we sought to answer, “How is our analysis responsive to epistemic ideas being tacit?” This paper can be read as our answers to this and similar questions.

### 2 Preventing a study from drifting far out of plumb requires consistent reflection throughout all phases of the work

Our study was constantly at risk of drifting out of plumb. When designing interviews, it was tempting to ask many decontextualized questions about the nature of good assessments (vs attempting to simulate authentic tasks). When analyzing our data, it was easy to fixate on explicit remarks about knowing and learning and neglect speech about other topics, even though a resources model of epistemic cognition posits that epistemic ideas are often underlying such speech. When we considered implications that could be drawn from our data, we sometimes drifted toward making sweeping statements about “better” and “worse” ways of thinking about assessing (vs considering responses as representing clusters of resources that seemed sensible to an instructor in a moment). While we do not claim our study is perfectly plumb, whatever coherence with theory we achieved was the result of many pointed discussions regarding how (or whether) our theory was showing up in our design, analysis, and implications.

### 3 Articulating how theoretical commitments show up across study activities provides useful context for others to engage with the work

The dynamics of how a study drifts away from or toward being plumb are an important part of the story of this study. Members of the broader community who have engaged with the study we describe here have been able to think more deeply about the claims we made as a result of understanding the theory underlying these claims as well as ways in which this theory informed all aspects of the study. We anticipate it would be useful for all members of our community to articulate ways in which they worked toward coherence with theory as they designed and carried out their studies. Doing so would make it clear what scholars assumed and allow readers to trace how these assumptions shape various study activities and implications drawn from these activities. As a diverse and thriving community, we have many different ways of modeling epistemology and most other theoretical constructs of interest. It is therefore unwise to assume everyone is in agreement about the finer points of a given theory.

In closing, we feel it is important to note that we do not intend for the takeaway message of this paper to be that our design and analysis decisions are the *best* or *only* way to study epistemologies. Our goal is not to standardize methods but rather to support our community in working to *align* theory and methods. We expect different researchers to use different methods and methodologies **depending on their theoretical frameworks**; we are advocating for researchers to carefully evaluate the congruency between the two when making their design and analysis decisions. This paper is one example of how such a careful evaluation might unfold. We hope this example helps others in our community reflect on how their theory and methods align.
